# Identification of differentially expressed HERV-K(HML-2) loci in colorectal cancer

**DOI:** 10.3389/fmicb.2023.1192900

**Published:** 2023-06-05

**Authors:** Qian Kang, Xin Guo, Tianfu Li, Caiqin Yang, Jingwan Han, Lei Jia, Yongjian Liu, Xiaolin Wang, Bohan Zhang, Jingyun Li, Hong-Ling Wen, Hanping Li, Lin Li

**Affiliations:** ^1^State Key Laboratory of Pathogen and Biosecurity, Department of Virology, Beijing Institute of Microbiology and Epidemiology, Academy of Military Medical Sciences, Beijing, China; ^2^Key Laboratory for the Prevention and Control of Infectious Diseases, Department of Microbiological Laboratory Technology, School of Public Health, Cheeloo College of Medicine, Shandong University, Jinan, China

**Keywords:** colorectal cancer, HERV-K, next-generation sequencing, differentially expressed loci, tumor immune

## Abstract

Colorectal cancer is one of the malignant tumors with the highest mortality rate in the world. Survival rates vary significantly among patients at various stages of the disease. A biomarker capable of early diagnosis is required to facilitate the early detection and treatment of colorectal cancer. Human endogenous retroviruses (HERVs) are abnormally expressed in various diseases, including cancer, and have been involved in cancer development. Real-time quantitative PCR was used to detect the transcript levels of HERV-K(HML-2) *gag*, *pol*, and *env* in colorectal cancer to systematically investigate the connection between HERV-K(HML-2) and colorectal cancer. The results showed that HERV-K(HML-2) transcript expression was significantly higher than healthy controls and was consistent at the population and cell levels. We also used next-generation sequencing to identify and characterize HERV-K(HML-2) loci that were differentially expressed between colorectal cancer patients and healthy individuals. The analysis revealed that these loci were concentrated in immune response signaling pathways, implying that HERV-K impacts the tumor-associated immune response. Our results indicated that HERV-K might serve as a screening tumor marker and a target for tumor immunotherapy in colorectal cancer.

## Introduction

Colorectal cancer (CRC), a malignant tumor that arises from the mucosa of the colon and rectum, is one of the deadliest cancers. According to IARC 2020 data, there were 1.93 million new CRC cases and 940,000 CRC deaths worldwide, placing the third in global cancer incidence and second in mortality (Sung et al., 2021). Colorectal cancer is predicted to rise to 2.5 million worldwide by 2035 (Arnold et al., 2017).

Colonoscopy is considered a sensitive and specific screening test for colorectal cancer, but it has limitations due to experience and patient compliance (Zygulska and Pierzchalski, 2022). Symptoms of colorectal cancer usually appear at an advanced stage. Risk factors for colorectal cancer include an aging population, poor diet, smoking, and obesity (Dekker et al., 2019). Statistical analysis showed that the 5-year survival rate of patients with stage I CRC was over 90%, while the 5-year survival rate of patients with stage IV CRC was only 10%(Brenner et al., 2014). Identifying molecular CRC markers is critical, as CRC is one of the most curable cancer types when detected early. Carcinoembryonic antigen (CEA), a clinically recognized colorectal cancer biomarker, is less sensitive and specific to the primary diagnosis. Studies have considered CEA as an independent prognostic factor and determined the impact of CEA surveillance on postoperative follow-up of CRC patients with stages II and III (Harrison et al., 1997; Duffy et al., 2003; Tsikitis et al., 2009; Thirunavukarasu et al., 2011). Recent works have demonstrated that genes associated with CRC have the potential to serve as a reliable predictor for cancer screening, treatment, and prognosis (Duffy et al., 2014; Lech et al., 2016). Unfortunately, the molecular role of these biomarkers in colorectal cancer remains to be further studied.

Human endogenous retroviruses (HERVs) are the remnant integrated into human germ cells by exogenous retrovirus infection and are inherited in a Mendelian fashion, accounting for about 8% of the human genome (Gifford and Tristem, 2003; Xing et al., 2013). HERVs are classified into three main groups based on the phylogenetic distinction of their exogenous ancestors: ERV-class I, ERV-class II, and ERV-class III. Most HERVs are transcriptionally silent in the host genome due to the accumulation of nonsense mutations, insertions, and deletions (Gifford and Tristem, 2003; Kassiotis, 2014). HERV-K belongs to the class II family and is the newly integrated, structurally complete, and biologically active HERV (Burn et al., 2022). HERV-K is generally repressed by epigenetic regulation, but under certain pathological conditions, it is aberrantly activated (Bock and Stoye, 2000). Several studies have shown that HERV-K is abnormally expressed in tumor tissues such as melanoma (Schmitt et al., 2013), breast (Wang-Johanning et al., 2001), prostate (Schulz, 2017), and liver (Ma et al., 2016). Furthermore, HERV expression correlates with tumor growth, metastasis, and overall survival, suggesting that HERV can be a diagnostic or prognostic cancer biomarker (Dervan et al., 2021). A previous study showed that HERV-W1, HERV-FRD1, HERV3-11, and HERV-V1 were overexpressed in the HCT8^WT/RETO^ colon carcinoma cell line resistant to etoposide chemical anticancer treatment. And the antiviral drugs down-regulated HERV gene expression, combined with anticancer drugs may have a synergistic antitumor effect, especially in chemorefractory tumors, which has therapeutic significance for CRC patients (Díaz-Carballo et al., 2015). HERV-H was transcriptionally upregulated in colorectal cancer tissue and was associated with mutations in several tumor suppressors, including ARID1A (Yu et al., 2022). In addition, the knockout of the HERV-K *env* affected the cell proliferation, migration, and tumor colony formation abilities of colorectal cancer cells through nupr1-related pathways (Ko et al., 2021). These results suggested a possible link between HERV and colorectal cancer. Most current studies above have focused on the expression of some elements of other HERV families and the HERV-K (HML-2) gene in colorectal cancer (Golkaram et al., 2021; Steiner et al., 2021; Yu et al., 2022). The relationship between HERV-K(HML-2) expressed in the blood of colorectal cancer lacks a more comprehensive description.

We selected blood samples from colorectal cancer patients and healthy individuals, colorectal cancer cells and control cell lines, then examined the differences in the transcript levels of HERV-K(HML-2) *gag*, *pol*, and *env* by Real-time quantitative polymerase chain reaction (RT-qPCR) to explore the association of HERV-K(HML-2) with the occurrence and development of colorectal cancer. Next-generation sequencing characterized the expression profile of HERV-K(HML-2) at differential expression loci in colorectal cancer patients and healthy individuals. The following pages discuss the potential of HERV-K (HML-2) as a new biomarker for early detection and diagnosis of colorectal cancer.

## Materials and methods

### Patients and samples

Blood samples were collected from 274 colorectal cancer patients. Regarding disease stage, there were 13 cases of stage I-II and 84 cases of stage III-IV. In addition, 147 age-and sex-matched individuals with no history of tumors, autoimmune diseases, or other diseases were selected as healthy controls. Written informed consent was obtained from all participants, and the data were analyzed anonymously. All blood samples were transported to the laboratory via cold chain and stored at −80°C. Demographic and basic information about the participants is presented in Table 1.

**Table 1:** Characteristics of the colorectal cancer patients and healthy controls.

Characteristics	Categories	Cases		Controls	
		*N* = 274	Mean ± SD	*N* = 147	Mean ± SD
Age (year)	20–40	19 (6.93%)	58.82 ± 11.22	36 (24.49%)	48.41 ± 14.32
	41–60	137 (50%)		79 (53.74%)	
	61–80	112 (40.87%)		31 (21.09%)	
	81–100	7 (2.55%)		1 (0.68%)	
Gender	Male	177 (64.6%)	–	99 (67.34%)	
	Female	97 (35.4%)	–	48 (32.65%)	
Disease stage	I-II	13 (4.74%)	–		
	III-IV	84 (30.66%)	–		

### Cell lines and culture

The human colon mucosal epithelial cell line, NCM460, and three CRC cell lines, HCT116, HT29, and SW480, were obtained from iCell Bioscience Inc. (Shanghai, China) and Procell Life Science Technology Ltd. (Wuhan, China), respectively. NCM460, HCT116, HT29, and SW480 were cultured in RPMI 1640 or DMEM with 10% FBS (Gibco, United States), 1% streptomycin/penicillin at 37°C, 5% CO2, and saturated humidity.

### RNA preparation and cDNA synthesis

Total RNA was extracted using the MiniBEST Universal RNA Extraction Kit (TaKaRa, Cat No. 9767) according to the manufacturer’s protocol. To remove genomic DNA, all RNA samples were treated with 1 μL of gDNA eraser per 1 μg of RNA (TaKaRa, Cat No. RR047A). DNA contamination of all RNA in the samples was detected using real-time quantitative PCR, which showed the CT value of β-actin was >40 in all samples. To further assess whether these gDNA-treated samples had any residual DNase activity or possible PCR inhibition, we added 10^3^ and 10^2^ DNA standards to the treated samples or pure water controls and compared their CT values. We also performed a double gDNA erasure treatment on low-copy RNA standards and found no evidence of RNA copy loss as the CT value did not change for successive treatments (Hurst et al., 2016; Karamitros et al., 2016). The RNA was then reverse transcribed into cDNA using a cDNA synthesis kit (TaKaRa, Cat No. RR047) according to the manufacturer’s protocol: 15 min at 42°C and 5 s at 85°C, followed by storage at 4°C.

### Real-time quantitative polymerase chain reaction for blood samples and cell lines

Real-time quantitative PCR was performed with a Roche LightCycler 480 II system in 96-well format, using MagicSYBR Mixture (Cwbio, Cat No. RR420A) in a 20 ul reaction with 2 μL cDNA, a final concentration of 0.25 μM forward primer and 0.25 μM reverse primer. All reactions were performed using the following protocol: cycling conditions were denaturation at 95°C for 10 min, followed by 40 cycles of 10 s at 95°C, 5 s at 60°C and 10 s at 72°C. The amplification and melting curves were analyzed at the end of the reaction. The presence of a single peak in the melting curve analysis was applied to verify the specificity of the PCR amplification. For each amplification, cDNA samples were taken in triplicate. Relative gene expression was determined by the 2^−ΔΔCT^ method, which normalized to a reference gene (*β*-actin) and compared to healthy controls. The primer sequences used to amplify HERV-K(HML-2) *gag*, *pol*, *env*, and β-actin were obtained from a previous study (Li et al., 2021). These primers have been identified to amplify the various loci of HERV-K (Yang et al., 2022). Primer information is shown in Table 2.

**Table 2:** Primers of this study.

Target gene	Primer sequence	Direction
β-actin F	CCACGAAACTACGTTCAACTCC	Forward
β-actin R	GTGATCTCCTTCTGCATCCTGT	Reverse
HERV-K(HML-2) *gag* F	AGCAGGTCAGGTGCCTGTAACATT	Forward
HERV-K(HML-2) *gag* R	TGGTGCCGTAGGATTAAGTCTCCT	Reverse
HERV-K(HML-2) *pol* F	TCACATGGAAACAGGCAAAA	Forward
HERV-K(HML-2) *pol* R	AGGTACATGCGTGACATCCA	Reverse
HERV-K(HML-2) *env* F	CTGAGGCAATTGCAGGAGTT	Forward
HERV-K(HML-2) *env* R	GCTGTCTCTTCGGAGCTGTT	Reverse

### Quantitative detection of HERV-K(HML-2) transcripts in colorectal cell lines by RNAscope® ISH technology

RNAscope® (RNAscope® Fluorescence Multiplexed reagent Kit, Advanced Cellular Diagnostics, United States) was according to the manufacturer’s protocol and is suitable for dual-detection mRNA. The probe set HERV-K(HML-2) *env*-C1, HERV-K(HML-2) *pol*-C2 or HERV-K(HML-2) *gag*-C3 (Advanced Cellular Diagnostics, United States) consists of 20 dual probes targeting different fragments within HERV-K(HML-2) *env*, HERV-K(HML-2) *pol*, or HERV-K(HML-2) *gag*. Standard samples were pretreated according to the manufacturer’s instructions and previously reported literature (Wang et al., 2012; Li et al., 2021). After treating the cells, they were inoculated on culture dishes. The slides were immersed in 10% neutral buffered formalin (NBF) and fixed at room temperature (RT) for 30 min. Pre-treatments for storage were carried out by dehydration with 50, 70, and 100% ethanol for 5 min at room temperature, followed by rehydration with 100, 70, and 50% ethanol at room temperature. Remove the slides from 1 × PBS and create a hydrophobic barrier using the Immedge ™ Hydrophobic Barrier Pen (Vector Laboratories, United States). The slides were then incubated with hydrogen peroxide and protease (Advanced Cell Diagnostics, United States) for 10 and 30 min at 40°C. Probes were mixed according to dose requirements. The mixed target probes were then incubated with the HyBez Incubation™ Oven (Advanced Cell Diagnostics, United States) for 2 h at 40°C. The corresponding probes were added drop by drop to the negative (RNAscope®3-plex negative control probe, Cat No. 320871) and positive (RNAscope®3-plex positive control probe-Hs, Cat No. 320861) controls. The signal was amplified by incubating one drop of AMP-1, AMP-2, and AMP-3 for 30, 30, and 15 min at 40°C using the HyBez ™ Oven. Each target nucleic acid was fluorescence-stained with Opal™ 520 (PerkinElmer, Cat No. FP1487001KT), Opal™ 570 (PerkinElmer, Cat No. FP1488001KT), and Opal™ 690 (PerkinElmer, Cat No. FP1497001KT) at 40°C C for 30 min and washed twice with wash buffer. The nuclei were stained with DAPI (Advanced Cell Diagnosis, United States) for the 30 s. Prolong Gold AntiFade Mountant (Prolong™, Invitrogen) prevented fluorescence quenching. Coverslips were then covered and photographed as soon as possible. Excitation/emission and pass wavelengths were set to 340–370/410–470, 460–480/490–530, 510–550/570–590, and 630–650/640–670 nm, respectively, for the detection of DAPI, Opal™ 520, Opal™ 570 and Opal™ 690. A confocal microscope (NIKON TI2-E; CRESTOPTICS X-LIGHT V3) was used to capture super-resolution images.

### Differential loci expression

HERV-K *gag*, *pol*, *env* in blood samples of colorectal cancer patients and healthy individuals were amplified by PCR with the same qPCR primers (Table 2). The products were purified and sent for next-generation sequencing (SinoGenoMax, China). The telescope provided by previous reports (Bendall et al., 2019) was used to identify HERV-K and quantify their expression from the sequencing data above. The sequenced reads of CRC were aligned to the sequencing results of healthy control using HISAT2 (Kim et al., 2015). The resulting SAM files were sorted and converted to BAM format using SamTools (Li et al., 2009). FeatureCounts were then used to obtain raw counts for transcripts aligned to the human genome’s HERV-K(HML-2) gene (Liao et al., 2014). The R package DESeq2 (v1.24.0) was used to evaluate differential gene expression on count values (Love et al., 2014). First, *P*adj-value<0.05 and |log2FoldChange| > 1 were the thresholds for identifying differentially expressed loci. Then *P*adj-value<0.05 and log2FC >0 were defined as “upregulated,” and *P*adj-value<0.05 and log2FC < 0 were described as “downregulated.” Use SangerBox to map the volcano plot (Shen et al., 2022). Crossover genes at differential sites were searched using bedtools (Quinlan and Hall, 2010). We used the library RIdeogram to construct the distribution location of different loci of HERV-K (HML-2) *gag*, *env*, and *pol* on human chromosomes. These different expressed loci functions were then analyzed using WebGestalt (Liao et al., 2019).

### Statistical analysis

Statistical analysis used GraphPad Prism 8 (GraphPad, United States) and SPSS version 25.0 (SPSS, United States). Shapiro–Wilk was applied to test for a normal distribution. When the data were normally distributed, independent group t-tests and one-way analysis of variance (ANOVA) were used to compare the mean ± SEM of tests obtained from at least three independent experiments; otherwise, Mann–Whitney test or the Kruskal–Walis test were used. *p*-values <0.05 were considered significant and *p < 0.05, * *p* < 0.01, ***p < 0.001 and ****p < 0.0001.

## Results

### Transcription of HERV-K(HML-2) *gag*, *pol*, and *env* in the blood of colorectal cancer patients by RT-qPCR

**Figure 1:**
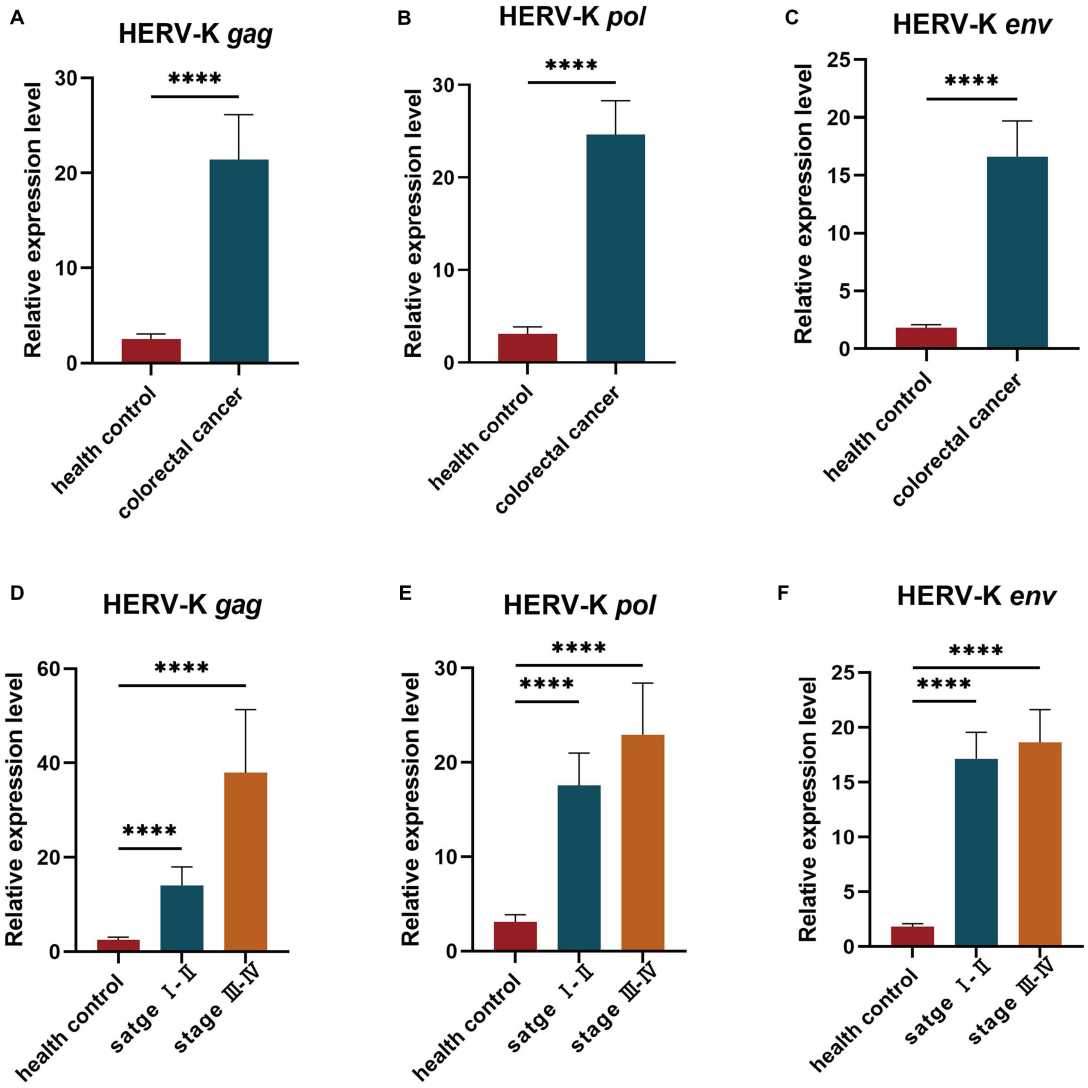
**(A–C)** The HERV-K (HML-2) *gag*, *pol*, and *env* gene expression in colorectal cancer patients and healthy individuals. **(D–F)** The HERV-K (HML-2) *gag*, *pol*, and *env* gene expression in colorectal cancer patients with stages I–II and III–IV. Experiments were repeated three times to ensure that the results were replicable. *p < 0.05, **p < 0.01, ***p < 0.001, ****p < 0.0001.

We collected background information and blood samples from 274 colorectal cancer patients and 147 healthy people. We then analyzed the transcript levels of HERV-K(HML-2) *gag*, *pol*, and *env* genes in colorectal cancer patients and healthy individuals by RT-qPCR. The transcript levels of HERV-K(HML-2) *gag*, *pol*, and *env* were significantly upregulated in colorectal cancer compared to healthy controls (Figures 1A–C). We also detected the expression of HERV-K(HML-2) genes in the blood of colorectal cancer patients with different stages. The results showed that HERV-K(HML-2) *gag*, *pol*, and *env* gene transcript levels were higher in colorectal cancer patients at different stages (I-II and III-IV) than in healthy controls. However, there was no significant difference in the expression of HERV-K among various disease stages (Figures 1D–F).

### Transcription of HERV-K(HML-2) *gag*, *pol*, and *env* among colorectal cancer cell lines by RT-qPCR

**Figure 2:**
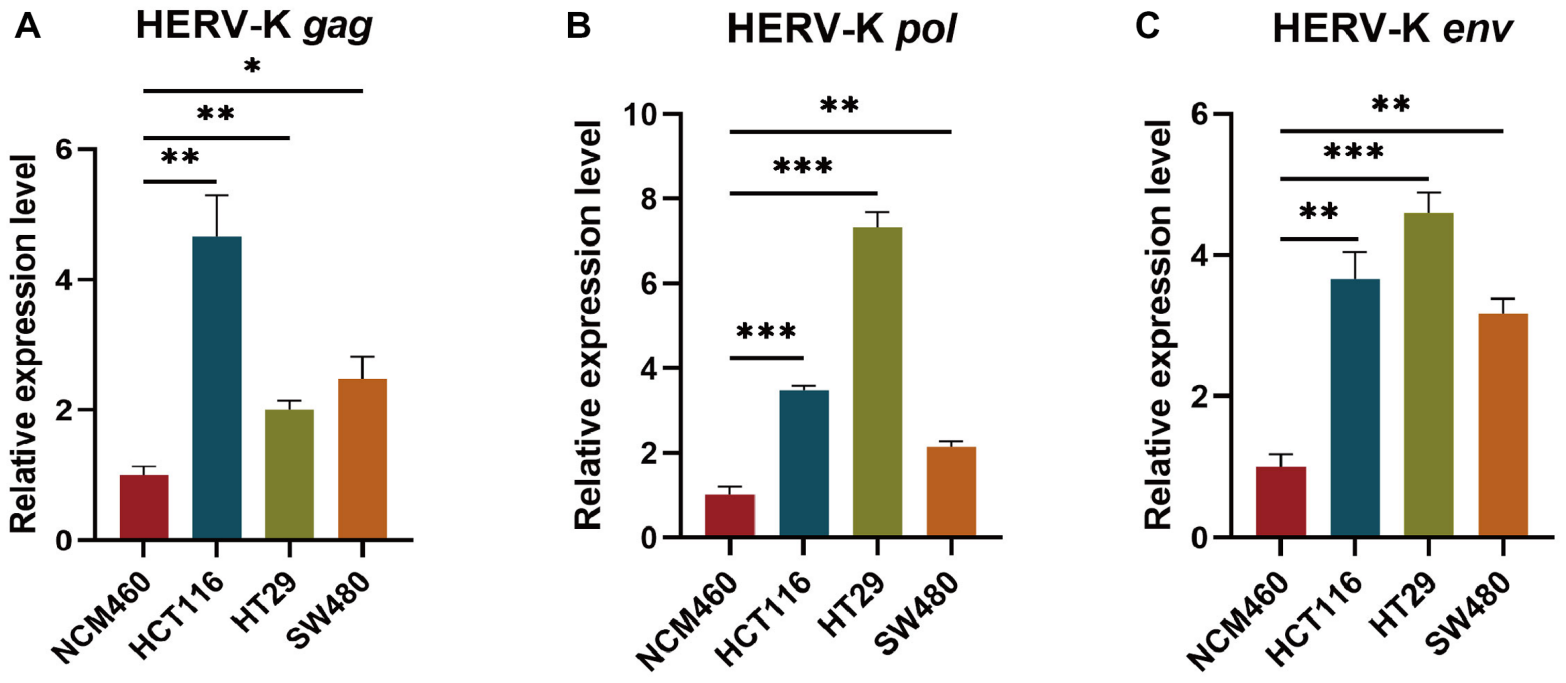
**(A–C)** Comparing the HERV-K (HML-2) *gag*, *pol*, and *env* gene in colorectal cancer cell lines HCT116, HT29, SW480, and the human intestinal epithelial cell NCM460. Experiments were repeated three times to ensure that the results were replicable. *p < 0.05, **p < 0.01, ***p < 0.001, ****p < 0.0001.

We examined the transcriptional levels of HERV-K(HML-2) *gag*, *pol*, and *env* mRNA in colorectal cancer cell lines HCT116, HT29, SW480, and NCM460 by RT-qPCR. Colorectal cancer cell lines had significantly higher HERV-K(HML-2) transcript levels of *gag*, *pol*, and *env* genes than healthy control (Figure 2).

### RNAscope® ISH technology assesses HERV-K(HML-2) *gag*, *pol*, and *env* expression in colorectal cancer cell lines

**Figure 3:**
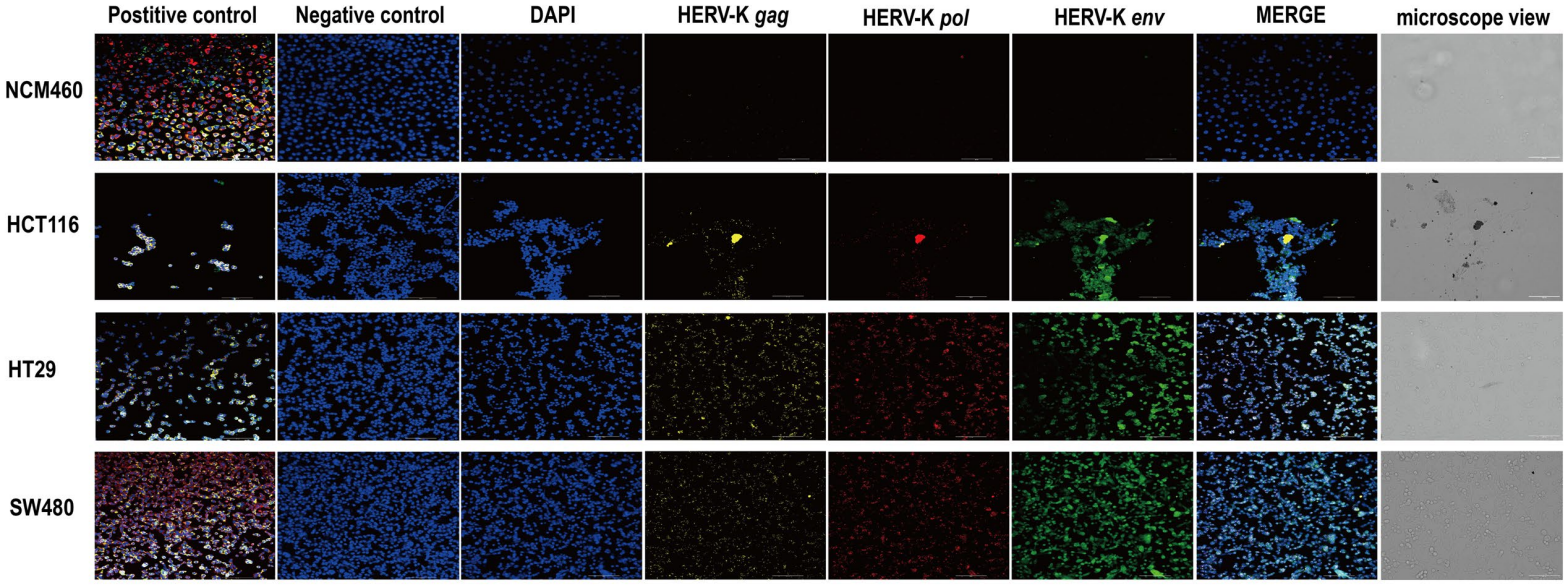
RNAscope® ISH analysis of HERV-K (HML-2) gag, pol, and env gene expression in colorectal cancer cell lines. The nuclei were stained and labeled with DAPI, HERV-K (HML-2) *env* region mRNA (green) was stained and labeled by Opal™ 520 fluorescent dye with a specific probe, HERV-K (HML-2) *pol* region mRNA (red) was stained and labeled by Opal™ 570 fluorescent dye with a special probe, HERV-K (HML-2) *gag* region mRNA (yellow) was stained and labeled by Opal™ 690 fluorescent dye with a unique probe. The standard control probes included in the kit (ACD) were used for positive and negative controls. Original magnification: 20×; scale bars: 100 μM.

We used HCT116, HT29, SW480, and NCM460 cells to show the RNA expression site and relative abundance in HERV-K(HML-2) *gag*, *pol*, and *env* in the situ with RNAScope® ISH technique. Sections were performed when cell density reached 60–70%. At the same time, positive and negative control sections were prepared. Compared to healthy controls, the transcriptional levels of HERV-K(HML-2) *gag*, *pol*, and *env* were significantly higher in colorectal cancer. Experimental results of RNAScope® were consistent with RT-qPCR results at the cellular level (Figure 3).

### Differentially expression loci analysis

**Figure 4:**
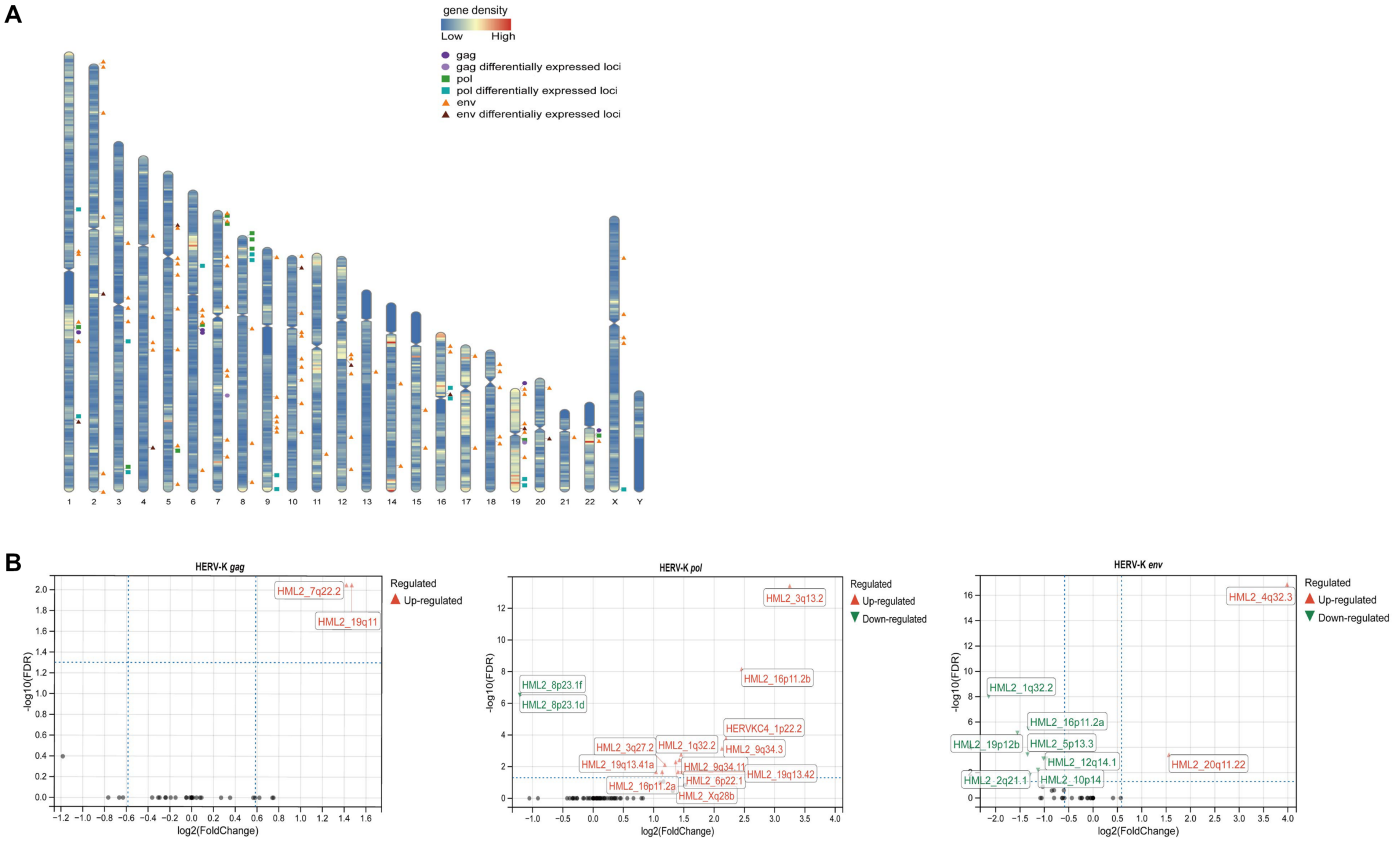
**(A)** Diagram of the distribution of HERV-K (HML-2) gag, pol, env and differentially expressed loci in the human genome. The circles represented HERV-K (HML-2) *gag* (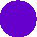) and its differential loci (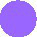), the squares represented HERV-K (HML-2) *pol* (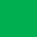) and its differential loci (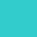), and the triangles represented HERV-K (HML-2) *env* (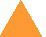) and its differential loci (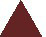). **(B)** Volcano plots derived from the HERV-K differential expression analysis, between colorectal cancer patients and healthy individuals. The up-regulated (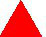) loci and down-regulated (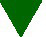) loci were marked. *P*adj-value<0.05 and |log2FC| > 1 were the thresholds for identifying differentially expressed loci.

After next-generation sequencing and screening of chromosome loci with 100% identity, we found that 5 HERV-K(HML-2) *gag*, 11 *pol* and 89 *env* loci were expressed in the human genome (Figure 4A). Then, we revealed different HERV-K(HML-2) loci expressed in colorectal cancer patients. There were 2 differential loci for the HERV-K(HML-2) *gag* (2 upregulated), 14 differential loci for the HERV-K(HML-2) *pol* (12 upregulated, 2 down-regulated), and 9 differential loci for the HERV-K(HML-2) *env* (2 upregulated, 7 down-regulated) in the blood samples of colorectal cancer patients (Figure 4B) (|log2FC| > 1.0, *P*adj-value < 0.05).

### Gene ontology annotation and KEGG pathway enrichment analyses of HERV-K(HML-2) differentially expressed loci

**Figure 5:**
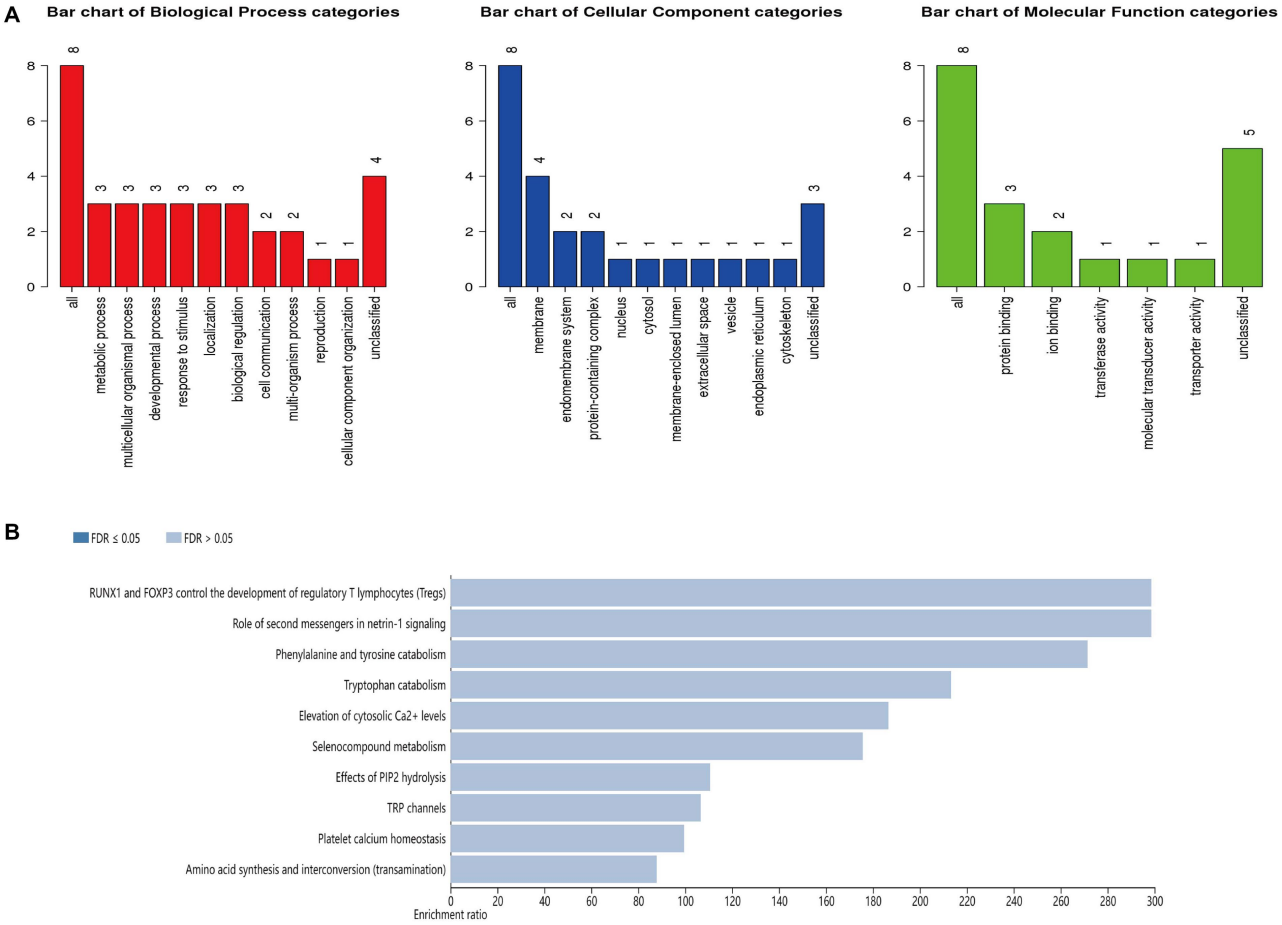
**(A)** GO functional classification of differentially expressed loci of HERV-K (HML-2). A red, blue, or green bar represented each biological progress, cellular component, and molecular function category. **(B)** The top 10 enriched KEGG pathways of differentially expressed HERV-K (HML-2) loci.

This study performed GO annotation and KEGG enrichment analyses to determine the function of all differentially expressed loci. WebGestalt was employed to conduct Gene Ontology (GO) annotation and Kyoto Encyclopedia of Genes and Genomes (KEGG) pathway enrichment analyses (Figure 5). GO biological process (BP) analysis of differentially expressed HERV-K(HML-2) loci revealed that these loci were significantly enriched in the metabolism process. The membrane was the top enriched GO cellular component (CC) analysis term. Protein binding was the most significantly enriched molecular function (MF) term. Besides, the top significantly enriched pathway for these loci was the RUNX1 and FOXP3 control of regulatory T lymphocyte development (Tregs).

## Discussion

Colorectal cancer has a complex pathogenesis and early nontypical symptoms that are difficult to detect without a sensitive and practical early screening test. The primary and most effective treatment for colorectal cancer is surgery. Nevertheless, the postoperative survival rate varies significantly due to the influence of the tumor stage. As a result, identifying an effective tumor biomarker that can be applied to early screening is critical to reducing colorectal cancer incidence and mortality. According to this study, the expression of the HERV-K(HML-2) *gag*, *pol*, and *env* was significantly higher in colorectal cancer patients and cell lines than in healthy controls. The RNAscope ®ISH technique was used to validate this result at the cellular level.

Detecting HERV-K in blood as a non-invasive molecular marker could provide a rapid and effective method for tumor screening and assessment (Dervan et al., 2021). Upregulation of HERV-K mRNA and encoded protein expression has been frequently reported in various cancers recently (Shah et al., 2022; Wei et al., 2022; Yang et al., 2022). Although its role in oncogenic mechanisms is still unidentified, it is clear that abnormal HERV-K expression can be used as a diagnostic and therapeutic target for cancer (Zhang et al., 2019). The expression of HERV-K *env*, *gag,* and *np9* mRNA in breast cancer tissue was increased, and its expression with treatment or not was a potential diagnostic marker for breast cancer (Tavakolian et al., 2019). In breast cancer cells, artificial regulation of HERV-K *env* expression may affect the expression of tumor-associated genes affecting cell proliferation, migration, and invasion, suggesting that HERV-K *env* proteins are essential for breast cancer development and metastasis (Zhou et al., 2016). A study concluded that HERV-K *env* mRNA could be a promising non-invasive blood biomarker to predict, detect, and monitor lung cancer (Zare et al., 2018). Moreover, patients with high HERV-K expression exhibited worse overall survival than those with moderate or low expression tumors, suggesting that HERV-K gene expression was associated with tumor size, stage, and lymph node metastasis. HERV-K transcripts were elevated in hepatocellular carcinoma (HCC) tissues compared with adjacent healthy tissues. The expression of HERV-K in HCC was related to TNM (Tumor Node Metastasis) stage and tumor differentiation, and the overall survival of patients with high expression was poor (Ma et al., 2016). HERV-K showed some potential diagnostic accuracy and may become a new candidate as a prognostic marker in HCC.

In addition, next-generation sequencing (NGS) technology has led to a more in-depth study of HERV and quantifying HERV expression in various samples. Many works have examined specific HERV-K loci to characterize their role in cancer. Analysis of HERV-K(HML-1) expression patterns at the level of individual proviral virions in the teratoma cell line Tera-2 revealed that HERV-K(HML-1) transcripts were predominantly derived from chromosome 22 (Bhardwaj et al., 2015). Besides, specific loci are associated with particular diseases. HERV-K (HML-2) upregulated in prostate cancer was limited to a few loci, notably 2q11.23 and 3q12.3 (Goering et al., 2011). The preferential expression of HERV-K *gag* 22q11.23 in prostate cancer tissues and the observed increase in advanced prostate cancer made this protein a potential biomarker for detecting prostate cancer progression and recurrence rates (Reis et al., 2013). The expression profiles of 91 known HERV-K proviruses could be used as biomarkers to distinguish breast cancer from healthy individuals (Wei et al., 2022). For example, HERV-K(HML-2) 17p13.1 provirus was strongly associated with breast cancer risk (Wei et al., 2022). Analyzing differentially expressed HERV in RNA-seq data from TCGA-COAD, results showed that upregulated genes enriched pathways related to epigenetic gene regulation. Four top 10 downregulated gene-enriched pathways were associated with immune responses and immunoglobulin cycling (Steiner et al., 2021). Also, genes associated with colon cancer were differentially expressed in these samples. Exploring HERV-K-specific loci in disease is vital for understanding the association of HERV-K with disease development.

This study characterized the differential expression profile of HERV-K *gag*, *pol*, and *env* loci in blood samples of colorectal cancer patients and healthy people by NGS. The analysis discovered that all differentially expressed HERV-K(HML-2) proviruses belonged to a separate LTR, and some of these HERV-K proviruses intersected with protein-coding genes and lncRNAs.

The HERV-K(HML-2) *gag* 7q22.2 was significantly upregulated in colorectal cancer. The LHFPL3 gene intersecting with HML_2 7q22.2 was a lipoma HMGIC fusion chaperone (LHFP) gene family member. LHFPL3 is involved in cell cycle control, and dysfunction of these genes may increase tumor susceptibility. It has been shown that the downregulation of LHFPL3 expression was associated with reduced viability, proliferation, and invasiveness of human glioma cells (Li et al., 2019). When the deletion occurs in LHFP3, its gene function is impaired, resulting in a worse prognosis for colorectal cancer patients (Lin et al., 2021). KYAT1 and CR1 are protein-coding genes intersecting with the upregulated differential loci of HERV-K (HML-2) *pol*. Kynurenine aminotransferase 1 (KYAT1) is critical in Se-methylselenocysteine (MSC) metabolism. KYAT1 exists in the cytoplasm and is a bifunctional enzyme with transamination and β-elimination activities (Selvam and Björnstedt, 2020). KYAT1 uses MSCs as substrates to generate two metabolites, MS and MSP, both of which have antitumor and antiproliferative properties (Rooseboom et al., 2001; Lee et al., 2009; Zeng, 2009). MicroRNA-guided tumor-specific induction of KYAT1 combined with the MSC has great potential in treating cancers beyond cure (Selvam et al., 2021). As part of the complement system, CR1 was a master regulator of complement activation, chronic inflammation, and innate immune responses (Jurianz et al., 1999). Chronic inflammation has been shown to trigger the development and spread of cancer (Coussens and Werb, 2002). Studies have shown a correlation between CR1 and certain malignancies. For example, the expression of CR1 mRNA was upregulated in nasopharyngeal carcinoma, and the risk of death in patients with high CR1 expression was significantly higher than that in patients with low CR1 expression (He et al., 2012). The potential interaction between SNP (rs7525160 G > C) of CR1 and smoking status suggested that CR1 rs7525160 G > C was significantly related to an increased risk of lung cancer (Yu et al., 2014). There were no protein-coding genes or lncRNAs that were crossed across with the HERV-K (HML-2) env elevated loci at 4q32.3 and 20q11.22. Furthermore, a few research has looked into the function of other differentially expressed loci. The 1q32.2 gene inside the CR1 intron was down-regulated in our study but upregulated in response to macrophage polarization toward anti-inflammatory (M2) phenotypes after IL-10 therapy (Russ et al., 2023). It indicated that the same loci were expressed differently in various diseases. Therefore, Investigating these loci and analyzing their impact on colorectal cancer will thus be the focus of our future research.

According to the KEGG analysis, the HERV-K (HML-2) differential expression loci are mainly enriched in the RUNX1 and FOXP3 control the development of regulatory T lymphocytes (Tregs) pathway. RUNX1 and FOXP3 are essential T cell immune regulators. RUNX1 retroviral transduction induced the phenotype of Th17 cells and participated in Th17 cells differentiation (Ono et al., 2007). Foxp3, a winged helix/forkhead family transcription factor, is a major regulator of Treg cell development and function, inducing various cancer cells. Foxp3 was expressed in T cells but suppressed in epithelial tumor cells (Mu et al., 2014). Foxp3 was detected in epithelial cells of breast and prostate cancer patients, speculating that inhibition of Foxp3 expression in epithelial tumor cells may be a mechanism for tumors to escape immune surveillance (Chen et al., 2013; Lal et al., 2013). The complex formed by the interaction of RUNX1 and FOXP3 proteins can induce the expression of Treg cell-associated genes and exert Treg cell suppressive activity. It has been shown that Foxp3 forms a complex with Runx1 and RORγt in Th17 cells to inhibit the production of the pro-inflammatory factor IL-17 (Zhang et al., 2008; Wong et al., 2011). Recent studies have suggested that RUNX1 and Foxp3 can synergistically inhibit cancer cell proliferation and apoptosis, suppressing the growth and spread of cancer cells by inhibiting metabolic pathways and Ras or other signaling pathways.

As a result, the biological functions of the HERV-K differentially expressed loci listed above are linked to tumor growth or immune response (Ruberto et al., 2022; Hosseiniporgham and Sechi, 2023). The HERV-K protein is expressed in various cancers and has been considered a tumor-specific antigen (Rycaj et al., 2015). HERV-K activates innate immune responses via RIG-I and Toll-like receptor pathways, stimulates B and T cells, induces antibody and cytotoxic T-cell responses, and has potential applications in cancer immunotherapy (Yu et al., 2012; Smith et al., 2018). It has been shown that the ability of HERV to modulate the immune system in cancer is related to oncogenic processes and anticancer defense. HERV-H *env* ISD was essential for tumor cell immune evasion, metastatic invasion, and adhesion, which could induce tumor cell epithelial-mesenchymal transition, increase CCL19 factor expression, recruit and expand pluripotent immunoregulatory CD271 cells (Kudo-Saito et al., 2014).

Human endogenous retroviruses antigen immunogenicity and upregulated expression profile in tumor tissue can be combined as a new target for tumor immunotherapy. In addition, it has been shown that the expression of HERV-K in colorectal cancer was associated with specificity of tumor localization and changes in DNA methylation of retrotransposable elements (Dolci et al., 2020a,b). Demethylating agents commonly used in cancer treatments cause retrotransposons to express hypomethylated levels *in vivo*. HERV-K-positive EVs levels were increased in colorectal cancer cell lines Caco2 and SK-CO-1 after treatment with decitabine. There was a link between EVs concentrations and reduced levels of IL-1β and mpx expression. It has been proposed that HERV-positive EVs may function as an immunomodulator in tumor progression. (Ferrari et al., 2019). The IFN-1 immune response mediated by dsRNA induction of multiple upregulated HERV transcripts has been identified as a critical anticancer mechanism of demethylation (Chiappinelli et al., 2017). This stimulation that generated bidirectional HERV transcripts formed dsRNA detectable by cellular PRRs, and caused type I and type III IFN responses in colorectal cancer cells, reducing the antitumor activity of DNA methyltransferase inhibitors (Vitiello et al., 2021). These dsRNAs can be assumed to be PAMPs for human PRR, stimulating innate immune responses through the TLR3 signaling pathway. TIP60 downregulated H3K9me3 expression, resulting in the reactivation of specific ERV elements (ERV-K), the induction of STING interferon responses and IRF7 expression (Rajagopalan et al., 2018). Consequently, tumors with high HERV expression exhibit significant activation of PRRs, TLR3, and MDA5, all of which play essential roles in the immune response.

In conclusion, HERV-K(HML-2) *gag*, *pol*, and *env* expression were upregulated in colorectal cancer, implying a link between HERV-K(HML-2) expression and colorectal cancer. We also considered HERV-K(HML-2) a possible colorectal cancer biomarker. Meanwhile, it was demonstrated that the role of HERV-K(HML-2) in cancer immunotherapy had a revealing effect on the anticancer treatment of colorectal cancer patients for the functional enrichment of differential HERV-K(HML-2) loci in the blood of colorectal cancer patients. Corresponding HERV-K(HML-2) immune agents for colorectal cancer can be established to evaluate its prospect as an immunotherapeutic target.

The fact that we are performing next-generation sequencing based on PCR amplification of the HERV-K(HML-2) gene in blood samples is a limitation of this study. Sample quality may influence sequencing results as well as differential site screening. Furthermore, due to the limited type of samples and the patients’ backgrounds provided by the sample source unit, we lack samples from patients with carcinoma *in situ* (CIS), adenomatous polyps, and colorectal malignant polyps, as well as those with definite disease stage. These samples are crucial for studying colorectal cancer progression. As a result, we intend to collect blood and possibly tissue samples, and detailed background information from patients with the aforementioned diseases to refine the research. Besides, the functions of most differential expression loci of HERV-K(HML-2) we obtained have not been clarified. We will investigate the role of these differential loci in colorectal carcinogenesis and development in greater depth in future studies.

## Data availability statement

The data presented in the study are deposited in the National Genomics Data Center (NGDC) database (https://ngdc.cncb.ac.cn/), accession number HRA004267.

## Ethics statement

The studies involving human participants were reviewed and approved by Beijing Institute of Microbiology and Epidemiology. The patients/participants provided their written informed consent to participate in this study.

## Author contributions

QK, LL, and HL contributed to the conception and design of the study. XG and H-LW contributed to sample collection. QK contributed to organizing the database, performing the statistical analysis, and writing the first draft of the manuscript. TL and CY developed the experimental techniques. LJ, YL, and XW contributed to the bibliographic survey. BZ and JH edited the manuscript. All authors contributed to the article and approved the submitted version.

## Funding

This study was supported by the State Key Laboratory of Pathogen and Biosecurity (AMMS).

## Conflict of interest

The authors declare that the research was conducted in the absence of any commercial or financial relationships that could be construed as a potential conflict of interest.

## Publisher’s note

All claims expressed in this article are solely those of the authors and do not necessarily represent those of their affiliated organizations, or those of the publisher, the editors and the reviewers. Any product that may be evaluated in this article, or claim that may be made by its manufacturer, is not guaranteed or endorsed by the publisher.
