# Supplementary material for: Identification of differentially expressed HERV-K(HML-2) loci in colorectal cancer
Source: Front Microbiol. 2023 Jun 5;14:1192900. doi: 10.3389/fmicb.2023.1192900 (PMC10277637; doi:10.3389/fmicb.2023.1192900)
Supplement: Supplementary file 1 [file Table_1.DOCX]

Supplementary Material

**Identification of Differentially Expressed HERV-K(HML-2) Loci in Colorectal Cancer**

**Qian Kang^1†^, Xin Guo^2†^, Tianfu Li^1^, Caiqin Yang^1^, Jingwan Han^1^, Lei Jia^1^, Yongjian Liu^1^, Xiaolin Wang^1^, Bohan Zhang^1^, Jingyun Li^1^, Lin Li^1*^, Hanping Li^1*^ and Hong-Ling Wen^2*^**

*** Correspondence:**

**Lin Li:** [**dearwood@sina.com**](dearwood@sina.com)

**Hanping Li:** [**hanpingline@163.com**](hanpingline@163.com)

**Hong-Ling Wen:** [**wenhongling@sdu.edu.cn**](wenhongling@sdu.edu.cn)

**Table S1. Differentially expressed HERVK(HML-2) loci.**

|  | **Locus** | **Chr** | **Start** | **End** | **Log2FoldChange** | **Padj Value** | **Intersected**  **Gene** | **Intersected**  **Gene Type** | **Intersected**  **Gene ID** |
| --- | --- | --- | --- | --- | --- | --- | --- | --- | --- |
| **HERV-K(HML-2) *gag*** | HML2_7q22.2 | chr7 | 104748902 | 104752819 | 1.465898018 | 0.008912 | LHFPL3  LHFPL3-AS1 | protein_coding  lncRNA | ENSG00000187416  ENSG00000226869 |
|  | HML2_19q11 | chr19 | 27637590 | 27646453 | 1.417682165 | 0.008912 | ERVK28  AC112702.1 | lncRNA  TEC | ENSG00000267696  ENSG00000279882 |
| **HERV-K(HML-2) *pol*** | HML2_3q13.2 | chr3 | 113024277 | 113033435 | 3.248027782 | 3.81E-14 | AC078785.1 | lncRNA | ENSG00000240057 |
|  | HML2_16p11.2b | chr16 | 34997026 | 34999771 | 2.455031954 | 6.77E-09 | None | NA | None |
|  | HML2_8p23.1d | chr8 | 12216461 | 12225988 | 1.210300824 | 2.98E-07 | None | NA | None |
|  | HML2_8p23.1f | chr8 | 12461223 | 12468498 | 1.210300824 | 2.98E-07 | ENPP7P6  AC068587.4 | transcribed_unprocessed_pseudogene  lncRNA | ENSG00000255549  ENSG00000283674 |
|  | HERVKC4_1p22.2 | chr1 | 89086283 | 89088619 | 2.196563799 | 0.000167001 | None | NA | None |
|  | HML2_6p21.1 | chr6 | 42893671 | 42903629 | 1.520889566 | 0.000717794 | AL035587.3 | lncRNA | ENSG00000288010 |
|  | HML2_9q34.3 | chr9 | 136780314 | 136789776 | 1.424895391 | 0.001692556 | None | NA | None |
|  | HML2_9q34.11 | chr9 | 128850236 | 128857457 | 1.424895391 | 0.003709324 | AL441992.3  KYAT1 | protein_coding  protein_coding | ENSG00000286112  ENSG00000171097 |
|  | HML2_Xq28b | chrX | 154608423 | 154615762 | 1.363520051 | 0.005042517 | None | NA | None |
|  | HML2_3q27.2 | chr3 | 185562548 | 185571727 | 1.185398862 | 0.007553372 | None | NA | None |
|  | HML2_1q32.2 | chr1 | 207635112 | 207639291 | 1.45503207 | 0.021349151 | CR1 | protein_coding | ENSG00000203710 |
|  | HML2_19q13.42 | chr19 | 53359095 | 53364791 | 1.401919106 | 0.021349151 | None | NA | None |
|  | HML2_16p11.2a | chr16 | 34412057 | 34414804 | 1.141263731 | 0.021349151 | None | NA | None |
|  | HML2_19q13.41a | chr19 | 52745023 | 52748339 | 1.039597086 | 0.021349151 | None | NA | None |
| **HERV-K(HML-2) *env*** | HML2_4q32.3 | chr4 | 164995688 | 165002916 | 3.984093748 | 1.40E-17 | None | NA | None |
|  | HML2_1q32.2 | chr1 | 207635112 | 207639291 | 2.142343216 | 1.02E-08 | CR1 | protein_coding | ENSG00000203710 |
|  | HML2_16p11.2a | chr16 | 34412057 | 34414804 | 1.326024349 | 3.33E-06 | None | NA | None |
|  | HML2_19p12b | chr19 | 22575022 | 22581759 | 1.55359011 | 7.22E-06 | None | NA | None |
|  | HML2_5p13.3 | chr5 | 30486653 | 30496098 | 1.345197867 | 0.000375706 | None | NA | None |
|  | HML2_20q11.22 | chr20 | 34127754 | 34136578 | 1.558245025 | 0.000385743 | AL031668.2 | lncRNA | ENSG00000287853 |
|  | HML2_12q14.1 | chr12 | 58327459 | 58336915 | 1.016956597 | 0.00079443 | None | NA | None |
|  | HML2_10p14 | chr10 | 6824179 | 6833641 | 1.130263605 | 0.006126548 | AL392086.2  LINC00707  AL392086.1  AL392086.3 | lncRNA  lncRNA  lncRNA  lncRNA | ENSG00000285988  ENSG00000238266ENSG00000285845  ENSG00000287277 |
|  | HML2_2q21.1 | chr2 | 129961965 | 129965044 | 1.302412486 | 0.015574906 | None | NA | None |

**Table S2. GO terms of differentially expressed HERVK(HML-2) loci.**

|  | GO Accession | Term | Overlap |
| --- | --- | --- | --- |
| Biological Process (BP) | GO:0008152 | metabolic process | 3 |
|  | GO:0032501 | multicellular organismal process | 3 |
|  | GO:0032502 | developmental process | 3 |
|  | GO:0050896 | response to stimulus | 3 |
|  | GO:0051179 | localization | 3 |
|  | GO:0065007 | biological regulation | 3 |
|  | GO:0007154 | cell communication | 2 |
|  | GO:0051704 | multi-organism process | 2 |
|  | GO:0000003 | reproduction | 1 |
|  | GO:0016043 | cellular component organization | 1 |
| Cellular Component (CC) | GO:0016020 | membrane | 4 |
|  | GO:0012505 | endomembrane system | 2 |
|  | GO:0032991 | protein-containing complex | 2 |
|  | GO:0005634 | nucleus | 1 |
|  | GO:0005829 | cytosol | 1 |
|  | GO:0031974 | membrane-enclosed lumen | 1 |
|  | GO:0005615 | extracellular space | 1 |
|  | GO:0031982 | vesicle | 1 |
|  | GO:0005783 | endoplasmic reticulum | 1 |
|  | GO:0005856 | cytoskeleton | 1 |
| Molecular Function (MF) | GO:0005515 | protein binding | 3 |
|  | GO:0043167 | ion binding | 2 |
|  | GO:0016740 | transferase activity | 1 |
|  | GO:0060089 | molecular transducer activity | 1 |
|  | GO:0005215 | transporter activity | 1 |

**Table S3. Summary of pathways enriched in KEGG mapping**

| **GeneSet** | **Description** | **Size** | **Overlap** | **Expect** | **Enrichment Ratio** | **P Value** | **Overlap ID** | **Database** | **User ID** |
| --- | --- | --- | --- | --- | --- | --- | --- | --- | --- |
| R-HSA-8877330 | RUNX1 and FOXP3 control the development of regulatory T lymphocytes (Tregs) | 10 | 1 | 0.003349 | 298.6 | 0.003345 | 1378 | Reactome | ENSG00000203710 |
| R-HSA-418890 | Role of second messengers in netrin-1 signaling | 10 | 1 | 0.003349 | 298.6 | 0.003345 | 7225 | Reactome | ENSG00000137672 |
| R-HSA-71182 | Phenylalanine and tyrosine catabolism | 11 | 1 | 0.003684 | 271.4545455 | 0.003679 | 883 | Reactome | ENSG00000171097 |
| R-HSA-71240 | Tryptophan catabolism | 14 | 1 | 0.004689 | 213.2857143 | 0.004681 | 883 | Reactome | ENSG00000171097 |
| R-HSA-139853 | Elevation of cytosolic Ca2+ levels | 16 | 1 | 0.005358 | 186.625 | 0.005348 | 7225 | Reactome | ENSG00000137672 |
| hsa00450 | Selenocompound metabolism | 17 | 1 | 0.005693 | 175.6470588 | 0.005682 | 883 | KEGG | ENSG00000171097 |
| R-HSA-114508 | Effects of PIP2 hydrolysis | 27 | 1 | 0.009042 | 110.5925926 | 0.009013 | 7225 | Reactome | ENSG00000137672 |
| R-HSA-3295583 | TRP channels | 28 | 1 | 0.009377 | 106.6428571 | 0.009345 | 7225 | Reactome | ENSG00000137672 |
| R-HSA-418360 | Platelet calcium homeostasis | 30 | 1 | 0.010047 | 99.53333333 | 0.01001 | 7225 | Reactome | ENSG00000137672 |
| R-HSA-70614 | Amino acid synthesis and interconversion (transamination) | 34 | 1 | 0.011386 | 87.82352941 | 0.011339 | 883 | Reactome | ENSG00000171097 |
